# Actionable Exomic Secondary Findings in 280 Lebanese Participants

**DOI:** 10.3389/fgene.2020.00208

**Published:** 2020-03-13

**Authors:** Nadine Jalkh, Cybel Mehawej, Eliane Chouery

**Affiliations:** Unité de Génétique Médicale, Faculty of Medicine, Saint Joseph University, Beirut, Lebanon

**Keywords:** NGS, exome, Lebanon, secondary findings, medically actionable diseases

## Abstract

The expanded use of NGS tests in genetic diagnosis enables the massive generation of data related to each individual, among which some findings are of medical value. Over the last three and a half years, 280 unrelated Lebanese patients, presenting a wide spectrum of genetic disorders were referred to our center for genetic evaluation by WES. Molecular diagnosis was established in 56% of the cases, as was previously reported. The current study evaluates secondary findings in these patients in 59 genes, linked to conditions mostly responsive to medical interventions, as per the ACMG guidelines. Our analysis allowed us to detect 19 pathogenic/likely pathogenic variants in 24 individuals from our cohort. Dominant actionable variants were found in 17 individuals representing 6% of the studied population. Genes associated with dominant cardiac diseases were the most frequently mutated: variants were found in 2.1% of our cohort. Genetic predisposition to cancer syndromes was observed in 1.07% of the cases. In parallel to dominant disease alleles, our analysis identified a recessive pathogenic disease allele in 2.5% of the individuals included in this study. Of interest, some variants were detected in different patients from our cohort thus urging the study of their prevalence in our population and the implementation, when needed, of specific genetic testing in the neonatal screening panel. In conclusion, here we report the first study estimating the actionable pathogenic variant load in the Lebanese population. Communicating current findings to the patients will enable them to benefit from a multi-disciplinary approach. Furthermore, tailoring the ACMG guidelines to the population is suggested, especially in highly consanguineous populations where the information related to recessive alleles might be highly beneficial to patients and their families.

## Introduction

Completed in 2003, the HGP represents one of the greatest achievements of the latest century. This international collaborative work enabled the implementation, few years later, of the high-throughput sequencing that is also known as NGS. With this tremendous development, the genetic information became accessible at a large scale. The genetic diagnosis was rendered faster and more efficient. However, many legal, ethical and social concerns were raised in regards to the use of this information. For this purpose, the ELSI program was founded in 1990 to address the problems arising from the HGP and to outlaw the discrimination against people on the basis of their genetic patrimony. The expanded use of NGS tests in genetic diagnosis and research shed the light on issues related to the interpretation of the massively generated data, among which incidental or secondary findings that are not related to the condition for which the patient was tested but are of a medical value. How to “judge” genetic variants? When and what to report? How to translate the useful and reliable findings into a concise and precise clinical report?

In November 2011, the ACMG appointed a working group on incidental findings in clinical exome and genome sequencing to make recommendations about responsible management of genomic data. The working group and external reviewers initially considered 56 genes to be linked to conditions that are most likely responsive to medical intervention. According to their policy statement, reporting these secondary findings enables physicians to consider appropriate follow-up and intervention for the patients and their family members who are at increased risk for these diseases (Green et al., 2013). The list was then updated in 2016 to include a total of 59 genes (Kalia et al., 2017).

In parallel, and in order to develop guidelines for the interpretation of sequence variants, a workgroup consisting of members from the ACMG, the AMP and the CAP was formed in 2013 and their recommendations in regards with variants interpretation, classification and terminology were published in 2015 (Richards et al., 2015).

Over the last three and a half years, 280 unrelated Lebanese patients, presenting a wide spectrum of genetic disorders were referred, to the Medical Genetics Unit (UGM) of Saint Joseph University (USJ). Here we report the frequency of secondary findings obtained from WES analysis of this cohort, across the newly revised list of 59 actionable genes based on ACMG recommendations.

## Materials and Methods

### Clinical Samples

From January 2015 to December 2018 patients with genetically heterogeneous disorders referred to our laboratory for diagnostic purposes, were included in our study. The most common features of the 280 patients were neurodevelopmental (in 39.5% of the cases), neuromuscular diseases (10%), metabolic and mitochondrial disorders (9.5%), renal disorders (5%), hearing disorders (3.5%), isolated epilepsy (3%), bone diseases (2%), leukodystrophy (2%), visual disorders (1%) and other rare diseases (24.5%) (Jalkh et al., 2019). Approval to conduct the study was obtained from the Ethics Committee of Saint Joseph University, Beirut, Lebanon (CEHDF 1440). A prior informed consent, allowing the communication of secondary findings to enrolled patients in addition to data publication has been obtained. Peripheral blood was then collected from each individual enrolled in this study and DNA was extracted using the salting out method (Miller et al., 1988).

### WES Analysis

Exon capture and sequencing: The exome was captured using the SureSelect Human All Exons, reagents (Agilent Inc.,^®^ Santa Clara, CA, United States) according to the manufacturer’s standard protocol. The concentration of each library was determined using Agilent’s QPCR NGS Library Quantification Kit (G4880A). Samples were pooled prior to sequencing with a final concentration of each sample equal to 10 nM. Sequencing was performed on the Illumina HiSeq2000 platform using TruSeq v3 chemistry.

Mapping and alignment: Reads files (FASTQ) were generated from the sequencing platform via the manufacturer’s proprietary software. Reads were aligned to the hg19/b37 reference genome using the Burrows-Wheeler Aligner (BWA) package v0.6.1 (Li et al., 2009). Local realignment of the mapped reads around potential insertion/deletion (Indel) sites was carried out with the Genome Analysis Tool Kit (GATK) v1.6 (McKenna et al., 2010). Duplicate reads were marked using Picard v1.62. Additional BAM file manipulations were performed with Samtools 0.1.18 (Li and Durbin, 2009). Base quality (Phred scale) scores were recalibrated using GATK’s covariance recalibration. SNP and Indel variants called using the GATK Unified Genotyper for each sample (DePristo et al., 2011). Variants were called using high stringency settings and annotated with VarAFT software 2.16 (Desvignes et al., 2018) containing information from dbSNP147 and gnomAD v2.11^1^. Variant interpretation was limited to the genes that occurred in the 59 medically actionable genes listed in the ACMG guidelines (Kalia et al., 2017).

Variants in these genes were filtered for only protein-altering variants, including truncating variants (stop gain/loss, start loss, or frameshift), canonical splice-site variants, inframe indels affecting protein-coding regions, variants within the intron–exon boundary (ten bases flanking the exonic boundaries), and missense variants based on frequency of occurrence in dbSNPv137 (<1%), ExAC/gnomAD v2.11 (<1%) and our in-house database (<1%) containing exome data of 300 exomes. We took into consideration the ACMG guidelines for diagnostic variant interpretation (Richards et al., 2015). However, we only included variants reported as pathogenic or likely pathogenic in ClinVar (accession date: November 2019), which is a public archive that reports the correlation between genetic variants and phenotypes, with supporting evidence. Last but not least and in order to minimize sequencing artifacts, we reassessed quality metrics of all retained variants and filtered out all variants that do not comply with the criteria published by Patel et al. (2014). In other words, we filtered out variants that had a read depth lower than 15, a genotype quality score lower than 20 then we kept all heterozygous variants with an alternate allele ratio (alt ratio) ranging between 0.3 and 0.7 and all homozygous mutated variants with an alt ratio above 0.85.

## Results

Over the last three and a half years, 280 patients, seeking genetic diagnosis by WES, were referred to our institution. All of these (patients or their legal guardians) wished to receive information on actionable secondary findings, unrelated to the condition for which genetic testing was performed. As per the ACMG guidelines, 59 genes, linked to conditions that are most likely responsive to medical intervention, were analyzed in our cohort of patients.

Coverage data showed that 96.3% of the coding regions for these genes were sequenced at a minimum depth of 20 reads.

**FIGURE 1:**
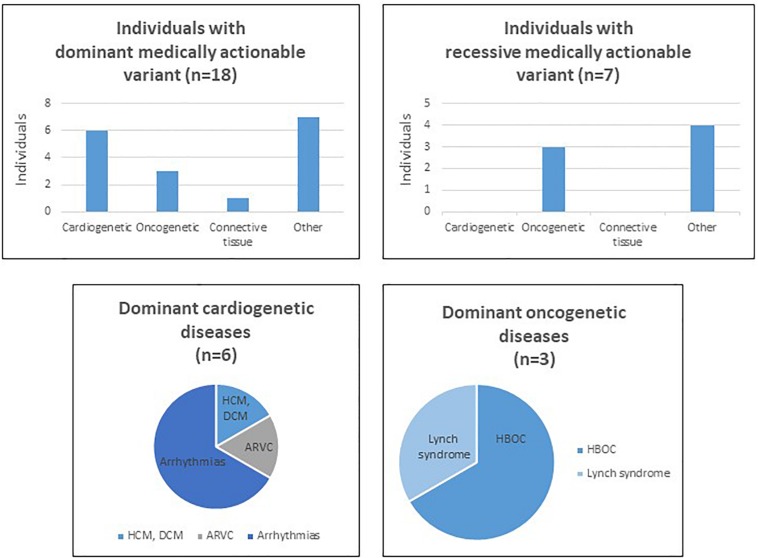
Charts and tables summarizing secondary findings in our cohort of patients. HCM, hypertrophic cardiomyopathy, DCM, dilated cardiomyopathy, ARVC, arrhythmogenic right ventricular cardiomyopathy.

WES allowed us to detect 4262 variants in the 59 selected genes, in our cohort of 280 individuals. Our filtering strategy enabled us to select in 24 individuals 19 variants that are reported as pathogenic or likely pathogenic in ClinVar (Table 1). The average of sequencing depth of the detected variants was 82x with a minimum of 20x coverage. We did not detect more than a variant in one individual; in other words, out of 280 individuals, 24 carry each a variant in one of the 59 medically actionable genes. Of these, 17 patients present a dominant medically actionable variant (6% of our cohort) and 7 are carriers of a high-risk recessive disease allele (2.5% of our cohort) (Figure 1 and Table 2). Some of the variants were found in a single gene, others in different individuals, which render the total number of mutated genes in our cohort equals 12: 10 linked to AD and 2 to AR diseases.

**TABLE 1:** Table summarizing secondary findings per disease type.

	Phenotype disease	Gene	Individuals
Cardiogenetic	Hypertrophic cardiomyopathy, dilated cardiomyopathy	*LMNA*	1
	Arrhythmogenic right ventricular cardiomyopathy	*TMEM43*	1
	Romano-Ward long QT syndromes 1, 2, and 3, Brudaga syndrome	*KCNQ1*	2
		*KCNH2*	2
Oncogenetic	Hereditary breast and ovarian cancer	*BRCA1*	1
		*BRCA2*	1
	Lynch syndrome	*PMS2*	1
	MYH-associated polyposis, Adenomas, multiple colorectal, FAP type 2, Colorectal adenomatous polyposis with pilomatricomas	*MUTYH*	3
Other	Familial hypercholesterolemia	*LDLR*	4
	Wilson disease	*ATP7B*	4
	Malignant hyperthermia susceptibility	*RYR1*	3
Connective tissue	Marfan’s syndrome	*FBN1*	1

**TABLE 2:** The ACMG list of the 59 actionable genes with the identified “pathogenic” or “likely pathogenic” variants in our cohort of patients.

MIM disorder	Typical age of onset	Gene	Inheritance	RefSeq ID	Nr of alleles identified	Pathogenic variants identified				ACMG-AMP classification	
							
						Genomic position	exon	cDNA position	Protein change	Conclusion	Categories
604370	Adult	*BRCA1*	AD	NM_007300.3	1	Chr 17: 41246531	exon10	c.1016dupA	p.K339fs	Pathogenic	PVS1 PM1 PM2 PP5 BS2
612555	–	*BRCA2*	AD	NM_000059.3	1	Chr 13: 32968825	exon25	c.9257-1G>A	–	Pathogenic	PVS1 PM2 PP3 PP5
151623	Child/Adult	*TP53*	AD	NM_001126112.2	–	–	–	–	–	–	–
175200	Child/Adult	*STK11*	AD	NM_000455.4	–	–	–	–	–	–	–
120435	Adult	*MLH1*	AD	NM_001258271.1	–	–	–	–	–	–	–
–	–	*MSH2*	AD	NM_000251.2	–	–	–	–	–	–	–
–	–	*MSH6*	AD	NM_001281493.1	–	–	–	–	–	–	–
–	–	*PMS2*	AD	NM_000535.6	1	Chr 7: 6026469	exon11	c.1927C>T	p.Q643X	Pathogenic	PVS1 PM1 PM2 PP5 BP4
175100	Child/Adult	*APC*	AD	NM_001127511.2	–	–	–	–	–	–	–
608456	Adult	*MUTYH*	AR	NM_001128425.1	2	Chr 1: 45797228	exon13	c.1187G>A	p.G396D	Likely Pathogenic	PS3 PM1 PP2 PP3
132600	–	–	–	–	1	Chr 1: 45797887	exon10	c.884C>T	p.P295L	Pathogenic	PS3 PM2 PM3 PP3 PP5
174900	Child/Adult	*BMPR1A*	AD	NM_004329.2	–	–	–	–	–	–	–
–	–	*SMAD4*	AD	NM_005359.5	–	–	–	–	–	–	–
193300	Child/Adult	*VHL*	AD	NM_198156.2	–	–	–	–	–	–	–
131100	Child/Adult	*MEN1*	AD	NM_130801.2	–	–	–	–	–	–	–
171400	Child/Adult	*RET*	AD	NM_020975.4	–	–	–	–	–	–	–
162300	–	–	–	–	–	–	–	–	–	–	–
1552401	Child/Adult	*RET*	AD	NM_020975.4	–	–	–	–	–	–	–
158350	Child/Adult	*PTEN*	AD	NM_000314.6	–	–	–	–	–	–	–
180200	Child	*RB1*	AD	NM_000321.2	–	–	–	–	–	–	–
168000	Child/Adult	*SDHD*	AD	NM_003002.3	–	–	–	–	–	–	–
601650	–	*SDHAF2*	AD	NM_017841.2	–	–	–	–	–	–	–
605373	–	*SDHC*	AD	NM_003001.3	–	–	–	–	–	–	–
115310	–	*SDHB*	AD	NM_003000.2	–	–	–	–	–	–	–
191100	Child	*TSC1*	AD	NM_000368.4	–	–	–	–	–	–	–
613254	–	*TSC2*	AD	NM_000548.4	–	–	–	–	–	–	–
194070	Child	*WT1*	AD	NM_001198551.1	–	–	–	–	–	–	–
101000	Child/Adult	*NF2*	AD	NM_000268.3	–	–	–	–	–	–	–
130050	Child/Adult	*COL3A1*	AD	NM_000090.3	–	–	–	–	–	–	–
154700	Child/Adult	*FBN1*	AD	NM_000138.4	1	Chr 15: 48748926	exon44	c.5330G>A	p.C1777Y	Likely Pathogenic	PM1 PM2 PP2 PP3 PP5
609192	–	*TGFBR1*	AD	NM_001130916.2	–	–	–	–	–	–	–
190182	–	*TGFBR2*	AD	NM_003242.5	–	–	–	–	–	–	–
613795	–	*SMAD3*	AD	NM_001145103.1	–	–	–	–	–	–	–
611788	–	*ACTA2*	AD	NM_001613.2	–	–	–	–	–	–	–
160745	–	*MYH11*	AD	NM_001040114.1	–	–	–	–	–	–	–
–	–	–	–	–	–	–	–	–	–	–	–
115197	Child/Adult	*MYBPC3*	AD	NM_000256.3	–	–	–	–	–	–	–
192600	–	*MYH7*	AD	NM_000257.3	–	–	–	–	–	–	–
601494	Child/Adult	*TNNT2*	AD	NM_001276346.1	–	–	–	–	–	–	–
613690	–	*TNNI3*	AD	NM_000363.4	–	–	–	–	–	–	–
115196	–	*TPM1*	AD	NM_001018008.1	–	–	–	–	–	–	–
608751	–	*MYL3*	AD	NM_000258.2	–	–	–	–	–	–	–
612098	–	*ACTC1*	AD	NM_005159.4	–	–	–	–	–	–	–
600858	–	*PRKAG2*	AD	NM_016203.3	–	–	–	–	–	–	–
301500	–	*GLA*	XLD	NM_000169.2	–	–	–	–	–	–	–
608758	–	*MYL2*	AD	NM_000432.3	–	–	–	–	–	–	–
115200	–	*LMNA*	AD	NM_170707.3	1	Chr 1: 156105714	exon6	c.959delT	p.R321fs	Pathogenic	PVS1 PM1 PM2 PP5
604772	Child/Adult	*RYR2*	AD	NM_001035.2	–	–	–	–	–	–	–
609040	Child/Adult	*PKP2*	AD	NM_004572.3	–	–	–	–	–	–	–
607450	–	*DSP*	AD	NM_001008844.2	–	–	–	–	–	–	–
610476	–	*DSC2*	AD	NM_024422.4	–	–	–	–	–	–	–
604400	–	*TMEM43*	AD	NM_024334.2	1	Chr 3: 14183192	exon12	c.1100G>A	p.G367D	Likely Pathogenic	PM1 PM2 PP3 PP5
610193	–	*DSG2*	AD	NM_001943.4	–	–	–	–	–	–	–
192500	Child/Adult	*KCNQ1*	AD	NM_181798.1	2	Chr 11: 2869089	exon16	c.1506delC	p.G502fs	Pathogenic	PVS1 PM1 PM2
613688	–	*KCNH2*	AD	NM_000238.3	1	Chr 7: 150644507	exon13	c.3060dupC	p.S1021fs	Pathogenic	PVS1 PM2 PP5
–	–	–	–	NM_172057	1	Chr 7: 150652569	exon1	c.23C>T	p.A8V	Likely Pathogenic	PS3 PM2 PP2 PP3 PP5
601144	–	*SCN5A*	AD	NM_198056.2	–	–	–	–	–	–	–
603830	–	–	–	–	–	–	–	–	–	–	–
143890	Child/Adult	*LDLR*	AD	NM_000527.4	3	Chr 19: 11231101	exon14	c.2043C>A	p.C681X	Pathogenic	PVS1 PM1 PM2 PP3 PP5
–	–	–	–	–	1	Chr 19: 11240211	exon17	c.2412delG	p.L804fs	Pathogenic	PVS1 PM1 PM2 PP5
–	–	*APOB*	AD	NM_000384.2		–	–	–	–	–	–
603776	–	*PCSK9*	AD	NM_174936.3	–	–	–	–	–	–	–
277900	Child	*ATP7B*	AR	NM_000053.2	1	Chr 13: 52511697	exon18	c.3818C>T	p.P1273L	Likely Pathogenic	PS3 PM1 PM2 PP3 PP5
–	–	–	–	–	1	Chr 13: 52515322	exon16	c.3451C>T	p.R1151C	Likely Pathogenic	PM1 PM2 PP3 PP5
–	–	–	–	–	1	Chr 13: 52509006	exon21	c.4283dupT	p.V1428fs	Likely Pathogenic	PVS1 PM2
–	–	–	–	–	1	Chr 13: 52531716	exon9	c.2383C>T	p.L795F	Likely Pathogenic	PM2 PP1 PP2 PP3 PP5
114208	Child/Adult	*CACNA1S*	AD	NM_000069.2	–	–	–	–	–	–	–
145600	–	*RYR1*	AD	NM_000540.2	2	Chr 19: 38937350	exon9	c.742G>C	p.G248R	Likely Pathogenic	PM1 PM2 PP2 PP3 PP5
–	–	–	–	–	1	Chr 19: 38964345	exon28	c.4094delG	p.G1365fs	Pathogenic	PVS1 PM2 PP5
311250	–	*OTC*	XLR	NM_000531.5	–	–	–	–	–	–	–

*AD, autosomal dominant; AR, autosomal recessive. PS, pathogenic strong, PVS, pathogenic very strong, PM, pathogenic moderate, PP, pathogenic supporting, BS, benign strong, BP, benign supporting.*

Among the dominant medically actionable variants, those associated with cardiac diseases were found in 6 individuals representing 25% of the patients with secondary reportable findings and 2.1% of our cohort. Variants in *KCNQ1* (NM_000218.2) or *KCNH2* (NM_000238.3) responsible for Romano-Ward long-QT syndrome types 1, 2, and 3 and for Brugada syndrome, were observed in 4 individuals.

Pathogenic variants in genes predisposing to hereditary cancer were detected in 3 individuals, representing 12.5% of the patients with secondary reportable findings and 1.07% of our cohort. An individual carried a pathogenic variant in *PMS2* (NM_000535.6) implicated in Lynch syndrome and the two patients carried each a variant in *BRCA1* (NM_007300.3) or *BRCA2* (NM_000059.3), associated with hereditary breast and ovarian cancer.

In addition to dominant disease alleles, 7 individuals, representing 29.1% of the patients with secondary reportable findings and 2.5% of our cohort, were found to be carriers of a high-risk disease allele in two recessive actionable genes (Figure 1). Pathogenic variants were observed in *MUTYH* (NM_001128425.1) in 3 individuals and in *ATP7B* (NM_000053.2) in 4 individuals. These genes are known to be involved in MUTYH Associated Polyposis and in Wilson disease, respectively. None of the 7 individuals carried homozygous or compound heterozygous recessive high-risk disease alleles.

## Discussion

Two hundred eighty Lebanese patients presenting with a wide range of genetic disorders were referred to our center for genetic diagnosis using WES. Molecular diagnosis was established for 56% of the cases (Jalkh et al., 2019). Furthermore, taking into consideration the ACMG guidelines, WES data was screened for variants in 59 medically actionable genes. Reporting secondary findings in these genes is indeed highly valuable to the individual and his/her family members, because it enables patients to benefit from an adequate clinical follow-up and management of the disease (McLaughlin et al., 2014). However, the interpretation of these findings should be made carefully since some pathogenic variants may be characterized by an incomplete penetrance or a variable expressivity. Early surveillance especially in the younger individuals is thus highly beneficial.

Our study allowed us to detect dominant actionable pathogenic or likely pathogenic variants in 6% of our Lebanese cohort. The frequency of secondary findings in the Lebanese population included in this study falls within the range of previously published studies (Xue et al., 2012; Cassa et al., 2013; Dorschner et al., 2013; Jang et al., 2015): while a study of 1,000 WES from European/African subjects yielded a rate of 1.2 to 3.4% of secondary findings (Dorschner et al., 2013), another study, by Xue et al. (2012) reported a frequency of 11% in 179 tested individuals. The variation in the rate of secondary findings between different groups is not surprising. Indeed, it depends on different factors among which the study design, the tested cohort, the sequencing technology and mainly data interpretation. The latter represents the biggest challenge in NGS studies since many variants remain to date “novel” or non-validated, they are classified as VUS and are thus unreportable in similar studies. Furthermore, incomplete penetrance and variable expressivity of a highly pathogenic variant might occur and render the interpretation of the data much complicated. Technical limitations should also be acknowledged as they can lead to missed variants such as the case of incomplete exome coverage or also the case of copy number variants (CNVs) that are not always included in WES analysis. Our study for instance did not include CNVs but only selected variants detected by WES and reported in ClinVar. We speculate that the development in the field of NGS technologies and data interpretation will lead to an increased rate of secondary findings in the near future.

That being said, our analysis revealed that genes associated with AD cardiac diseases were the most frequently mutated in our cohort. Early surveillance and intervention are critical to decrease the risk of sudden death for the concerned patients.

Secondary findings associated with cardiac conditions were identified in 6 individuals included in this study. Among these, four patients present a truncating variant in *KCNQ1* or *KCNH2*, predicted to be linked to the LQTS which is an inherited cardiac arrhythmic syndrome. The prevalence of the latter is estimated to be 1:2500 (Steffensen et al., 2015). However, its prevalence in the Lebanese population herein reported is 1:70 (4 out of 280), which represents a 35-fold increase in the prevalence in our cohort. Including additional patients in our study is crucial in order to better estimate the prevalence of this disease in our population. The same mutations in *KCQN1* and *KCNH2* were found in different patients, originated from different geographical areas and belonging to different religious subpopulations. Since these variants do not occur in a hot spot mutational site, one might question if they has arisen from a unique and very old mutational event. Studying the frequency of these variants and assessing the incidence of sudden death in the Lebanese population are crucial to determine whether the inclusion of specific genetic testing in the neonatal screening panel is needed.

Further to these 4 patients, 4 individuals have pathogenic truncating variants in *LDLR*, a gene associated with the most frequent form of AD familial hypercholesterolemia (FH). Of note, the incidence of FH is particularly high in the Lebanese population presumably due to a founder effect (Abifadel et al., 2009). Indeed, a study performed by Abifadel and colleagues have shown that the p.Cys681^∗^ mutation in the *LDLR* gene accounts for 81.5% of their FH studied Lebanese probands. Our data confirm the high prevalence of FH in the Lebanese population (1/70) compared to its prevalence in other populations (estimated between 1/200 and 1/500) (Benn et al., 2012; Nordestgaard et al., 2013). These findings raise the importance of establishing rapid and early screening methods for the molecular diagnosis of FH in our population in order to decrease coronary heart disease events and mortality.

In addition to secondary findings associated with cardiac diseases, genetic predisposition to cancer syndromes was observed in 3 individuals. Pathogenic *BRCA1/2* variants were found in 2 individuals representing 0.7% of our cohort. Breast cancer represents the most common cancer type in females and it constitutes one-third of all reported cancer cases, in Lebanon (Jalkh et al., 2017). Its incidence rate was projected to approach 137 per 100,000 (0.13%) in 2018 (Shamseddine et al., 2014). In parallel, the overall prevalence of *BRCA1/2* mutations is estimated to range between 1 in 400 (0.25%) to 1 in 800 (0.12%) but it varies depending on ethnicity (Petrucelli et al., 2010). The incidence herein reported shows a 10 time fold increase. However, this rate is not conclusive since the inclusion of additional individuals is needed for a better estimation of the prevalence of a disease in a population. One pathogenic variant in *PMS2* associated with Lynch syndrome was also identified in this study. This syndrome represents the most common cause of inherited colorectal cancer and is also characterized by common development of extracolonic malignancies, including cancers in the endometrium, ovaries, gastrointestinal tract, and urinary tract. Owing to the complicated nature of this disease, a multi-disciplinary approach, including genetic counseling, is needed for the management of these patients.

In addition to dominant disease alleles, our analysis identified 7 individuals with a recessive pathogenic disease allele, representing 2.5% of our cohort. Variants in *MUTYH* were detected in 3 individuals (in 1/90 of our cohort), and in the *ATP7B* gene in 4 individuals (in 1/70 of our cohort). The prevalence of heterozygous germline *MUTYH* and *ATP7B* variants in our cohort is comparable to that reported in the general population (1/100 and 2/100 for *MUTYH* and 1/90 for *ATP7B* variants) (Nielsen et al., 1993; Al-Tassan et al., 2002; Cleary et al., 2009; Win et al., 2017; Roberts, 2018). Since the guidelines of the ACMG recommend to only return bi-allelic pathogenic variants, carriers’ status was checked in our study but was not reported. Nevertheless, reporting carrier status might be highly beneficial especially in our population that is characterized by a high rate of consanguineous marriages (estimated to range between 15 and 35.5% depending on the community) (Khlat, 1988; Barbour and Salameh, 2009).

In conclusion, the tremendous development witnessed in the sequencing field, mainly in NGS techniques, goes along with the generation of massive genetic data related to each tested individual. Our mission as geneticists and scientists is to ensure the use of the available information in the best way that could serve the patient. As per the ACMG recommendations and owing to the importance of reporting secondary findings for the management of patients, a related informed consent was obtained from 280 patients who were referred to our center for genetic diagnosis. WES data was analyzed based on the list of the 59 “ACMG” genes, linked to conditions that are most likely responsive to medical intervention. Our study is the first to estimate the actionable pathogenic variant load in the Lebanese population. Reporting these findings to the patients will enable them to benefit from a multi-disciplinary approach, including a professional genetic counseling. This should also involve a detailed personal and family history, information on the disorder and genetic tests, discussion of the management and surveillance of the disease, career plan, family plan, and psychosocial support. Furthermore, the estimation of the prevalence of some diseases in specific populations might lead to the generation of “tailored” actionable genes’ lists and will help to consider implementing novel neonatal screening tests for the diseases that respect the “Wilson and Jungner criteria” (Andermann et al., 2008).

## Data Availability Statement

The datasets for this article are not publicly available because, per our ethical board requirements, we are not allowed to deposit all patients’ NGS data in a public repository since the consent form obtained in this study does not include this disclosure. Requests to access the datasets should be directed to the corresponding author.

## Ethics Statement

Written informed consent for participation and publication of the data was obtained from participants or from their legal guardian/next of kin in case participant is under 18 years of age.

## Author Contributions

NJ, CM, and EC conceived, designed the study, performed the data interpretation, and wrote the manuscript. NJ performed bioinformatics data analysis. All authors have read and approved the manuscript.

## Conflict of Interest

The authors declare that the research was conducted in the absence of any commercial or financial relationships that could be construed as a potential conflict of interest.

## Footnotes

1 https://gnomad.broadinstitute.org/

## Abbreviations

ACMG: American college of medical genetics and genomics
AD: autosomal dominant
AMP: association for molecular pathology
AR: autosomal recessive
BP: benign supporting
BS: benign strong
CAP: college of American pathologists
CNVs: copy number variations
ELSI: ethical legal and social implications program
HGP: human genome project
LQTS: long QT syndrome
NGS: next generation sequencing
PM: pathogenic moderate
PP: pathogenic supporting
PS: pathogenic strong
PVS: pathogenic very strong
UGM: medical genetics unit of saint Joseph University
VUS: variants of unknown significance
WES: whole exome sequencing.
